# An Export-Marketing Model for Pharmaceutical Firms (The Case of Iran)

**Published:** 2013

**Authors:** Mehdi Mohammadzadeh, Narges Aryanpour

**Affiliations:** Department of Pharmacoeconomics and Pharmacy Management, School of Pharmacy, Shahid Beheshti University of Medical Sciences. 14155-6153, Tehran, Iran.

**Keywords:** Internationalization, Export marketing, Pharmaceutical, Export readiness, Iran

## Abstract

Internationalization is a matter of committed decision-making that starts with export marketing, in which an organization tries to diagnose and use opportunities in target markets based on realistic evaluation of internal strengths and weaknesses with analysis of macro and microenvironments in order to gain presence in other countries. A developed model for export and international marketing of pharmaceutical companies is introduced. The paper reviews common theories of the internationalization process, followed by examining different methods and models for assessing preparation for export activities and examining conceptual model based on a single case study method on a basket of seven leading domestic firms by using mainly questionares as the data gathering tool along with interviews for bias reduction.

Finally, in keeping with the study objectives, the special aspects of the pharmaceutical marketing environment have been covered, revealing special dimensions of pharmaceutical marketing that have been embedded within the appropriate base model. The new model for international activities of pharmaceutical companies was refined by expert opinions extracted from result of questionnaires.

## Introduction

Given the fact that we remain in the infancy period of pharmaceutical marketing, the lack of literature in this field is not unforeseen. On the other hand, the quality of pharmaceutical marketing is significantly different from other types such as consumer marketing. This is mainly due to the differences in the nature of pharmaceutical products and the industrial atmosphere of pharmaceuticals, which have specific differences from that of other consuming goods. These deviations root in their particular regulatory restrictions, operational stage, and the health care environment. Therefore, from a theoretical perspective, this field can be considered as a subset of marketing science, sharing general principles, and holding specific individual inter-industry considerations at the same time (1). 

This study consists of three main sections. In the first section, viewpoints on export marketing and internationalization have been studied. Definitions, history, and various approaches have been included. The export marketing and readiness assessment theories, views, and relevant models and issues have been reviewed in the second part in order to elaborate differences among current models and their applicability in pharmaceutical companies. This is followed by the final part that signifies the characteristics and special aspects of the pharmaceutical marketing environment with the objective of developing an applicable model for export marketing and internationalization.

## Experimental

*Methodology*

The study was based on the development of a self-administered survey. First, the Cavusgil and Zou (2) instrument was incorporated into a preliminary questionnaire and was pretested via a series of personal interviews with the marketing managers of 7 Iranian pharmaceutical firms involved in exporting. Following some minor refinements, the questionnaire was sent to sample firms.

At least, ten experts in each of the firms have been interviewed or filled the questionnaire and asked respondents to define effective factors in aspects of firm and market that should consider in pharmaceutical marketing.

The impervious and filling the questionnaire was done during the pre-appointed personal meetings and explaining the questionnaire before being filled. We used the questionnaire to gather the information from the key persons and management teams of the firms and conducted several interviews with some of them and also some other opinion leaders in the healthcare system.

*Sample and data collection*

The sample consisted of seven Top Iranian Pharmaceutical firms has been selected for the reason that they have the sum of 40% of the domestic market share and sum of 70% of whole pharmaceutical exports (3).

*Data analysis*

A Factor analysis using the principal components method with Varimax rotation was conducted to identify the salient factors influencing the export marketing performance of Iranian Pharmaceutical firms exporting affairs.

Six dimensions for market-side and five dimensions for firm-side of Whitelock model as the conceptual model of this study were initially identified.

A test of reliability for the identified dimensions has resulted a Cronbach’s alpha of 0.85, 0.83, 0.81, 0.76, and 0,86 respectively, for the items “Orientation and Philosophy, Regulatory affairs skill, Recourses, Objectives and Management commitment” in firm-side. Items of “Healthcare system, Regulatory system, Influencers, Accessibility, Competitors and potential or Attractiveness of target market” in market-side of the model have resulted values of 0.84, 0.85, 0.79 and 0.8, respectively.

## Results

**Figure 1:**
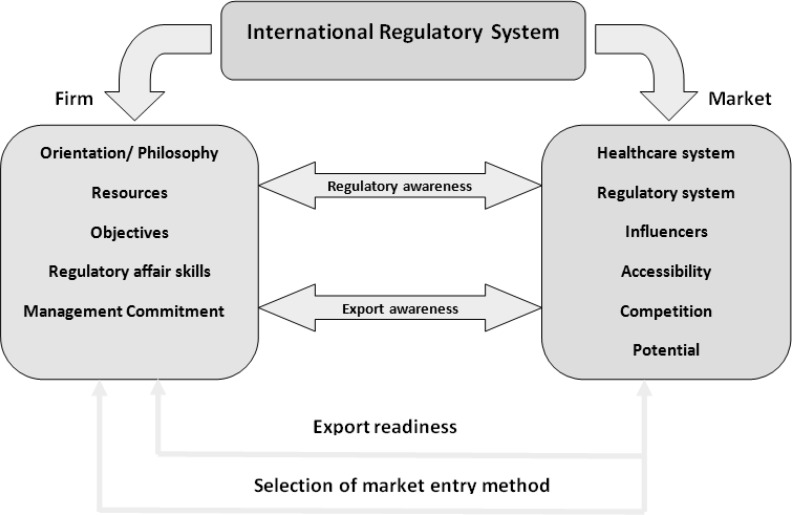
The developed model for internationalization tailored for pharmaceutical firms

After evaluating the above-mentioned theories and models, and analysis of data with considering the pharmaceutical marketing context, the influential and relevant factors that affect pharmaceutical export marketing were extracted and developed into a theoretical model as presented in Figure 1.

After analyzing the experts’ responses gathered from questionnaire and open-ended interviews, it was suggested that as the study is pertained to the pharmaceutical field, the components are enough to establish a new model based on the previously described Whitelock model for Iran. Several aspects which are further explained were considered to be embedded in the model as an alternative to some of the existing parameters.

Based on the results, the internal properties of the firm should be considered in light of the five dimensions as follows: orientation and philosophy, regulatory affairs skills, resources, objectives and management commitment. Meanwhile, the market-side of the model should incorporate the following six dimensions: healthcare system, regulatory system, influencers, accessibility, competition and potential or attractiveness of target market.

## Discussion

Internationalization is a matter of committed decision-making, in which an organization tries to diagnose and use opportunities in target markets on the basis of realistic evaluation of internal strengths and weaknesses in order to gain presence in other countries (4). As defined by Doole and Lowe, at its simplest level, internationalization involves the firm making marketing mix decisions in one or more targets across national boundaries and at its most complex level, involves establishing, manufacturing and processing the facilities worldwide and coordinating marketing strategies across the globe (5).

Furthermore, Doole and Lowe addressed different levels of involvement in international marketing as *export marketing*, providing goods or services across national and political borders, *International marketing, *marketing activities, interests, or operations in more than one country and *Global marketing, *focusing on selection and exploration of global marketing opportunities.

Internationalization has been explained in many theories and models by earlier researchers. The well-known theories can be classified into the following major theoretical models.

*Uppsala Model of Internationalization*

The Uppsala model seeks to explain how firms gradually intensify their activities in foreign markets. Based on this model, the main features of the Uppsala model is as follows: 1) firms begin with the domestic market before they move to foreign markets; 2) firms start foreign operations in culturally and/or geographically close countries and eventually enter the other markets that were further away in culture and/or geographical terms; 3) firms start their foreign operations by using conventional exports and gradually move towards using more intensive and demanding operation modes such as using their own sale organization, at the company and target country levels.

The Uppsala model also shows that the internationalization is a gradual developing process through which every firm takes certain consecutive steps; these normally start with exporting and progress through licensing, joint ventures, direct investment, and establishment of company owned sites across borders (6).

*Eclectic paradigm and transaction cost analysis theory*

The eclectic paradigm tries to explain the production on an international basis, which includes the ownership, location and internalization advantages while maintaining production abroad. This theory is a further development of internalization theory which itself is based on the transaction cost theory. The transaction cost theory tries to explain why companies exist, and why companies expand or source out activities to the external environment. Regarding this theory, the decision to enter international markets is made based on the firm’s goal for minimizing costs.

This theory mainly explains the firm’s procedure of decision-making (mainly multinational companies), on establishing the manufacturing subsidiaries in foreign markets (7). According to Anderson *et al.*, this approach begins with the assumption that the market is competitive and that there are many competitors in it (8, 1).

*Industrial networks*

Developed by the International Marketing and Purchasing (IMP) Group, Industrial networks theory describes the industrial system as a network of companies with lasting relationships in production, distribution, and use of goods together; furthermore, it highlights the important competitive relations and simultaneously individual relationships with external stakeholders such as distributors, suppliers and customers (9).

In contrast to the independence of international marketing decisions, which is elliptical in the Uppsala and Transaction Cost models, the Industrial Network approach model tries to study the behavior of the company as a member of an interdependent group. Indeed, in the Uppsala and Transaction Cost models, the characteristics of the firms and their markets have been omitted (10).

The main weakness of above-mentioned theories is that, they consider only the producer and the intermediary, play important roles in the flow of products or services. Whereas, the International Network approach explains the interaction of the network within which the firm is embedded and by that, determines the behavior of the firm in the market (9).

*Business strategy approach*

Welford and Prescott argue that the business strategy approach is based on the idea of pragmatism, with the firm making trade-offs between a number of variables in its decision to internationalize and the methods it adopts to do so (11).

Furthermore Turnbull discusses the factors, which should be evaluated using this approach, for market selection including Market attractiveness, Psychic distance, Accessibility, and Informal barriers (12).

In addition to these factors, they discuss the choice of organizational structure to serve the market as also being dependent on specific company factors such as International trading history, Company size, and Export orientation and commitment.

The above theories seem to indicate a diversity of opinion on the internationalization process of the firm, and provide different emphases on the issue of market entry and selection of its mode. The Uppsala model originally concentrated on the impact of one explanatory variable on this issue, namely, the firm’s experiential knowledge.

More recently, Johanson and Vahlne have acknowledged the importance of another variable, said to be implicit from the outset of relationships to other bodies (customers, suppliers, and competitors) in the foreign market (4).

The eclectic paradigm, rather than adopting such a behavioral approach to internationalization, focuses on a combination of economic theories of monopolistic competition, location, and transaction costs and assumes that a rational, optimal choice will be made. However, such an approach itself depends on “perfect’’ information.

Finally, the business strategy approach suggests that international activity is contingent on a range of variables related both to the specific firm and to the environment which it operates. These variables may themselves be a function of the perceptions and experience of the relevant decision makers within the firm.

*Whitelock model*

**Figure 2:**
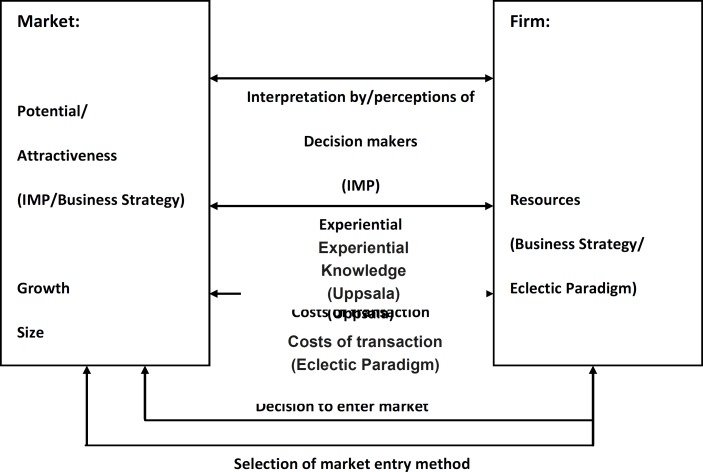
Whitelock model

Whitelock Model is an attempted integration of the key elements of each theory that is depicted in Figure 2. Whitelock concludes ”It would seem that, whilst acknowledging the distinctive contributions of each school of thought, a number of areas of convergence are also apparent. A model incorporating the key elements of each approach may present a more realistic and comprehensive picture of the market entry decision” (15).

*Market and the firm*

This model shows that how each of the previous models relates to the others and how it entails the two sections of the newly formed model.

*Export readiness models and theories*

Based on the theories discussed in the previous section, the international business of the domestic-born firms usually begins with export. Different models and assessment methods have been developed regarding export activities; those that have contributed to this study include Porter’s Diamond Model and Rahman Model.

*Porter’s Diamond model*

To understand the competitive advantage of nations, Porter offers the “Diamond Model”, which seeks to explain why particular industries become competitive (13). He introduced the concept of clusters or groups of interconnected firms, suppliers, related industries and institutions that arrive in particular locations (14).

The diamond model consists of six factors including factor conditions, demand conditions, related and supporting industries, firm strategy (structure and rivalry), government and chance.

The concept of the model is to encourage or even push companies to raise their aspirations and move to higher levels of competitive performance. They must encourage companies to raise their performance, stimulate early demand for advanced products, and focus on specialized factor creation. Furthermore, they must stimulate local competition by limiting direct cooperation and enforcing anti-trust regulations.

*Rahman Model*

Rahman model focuses on an effective international positioning process. It describes four phases for international business as follows (14):

Phase one: Assesses the international business capability of an organization.

Phase two: Analyzing the external environment to identify the potential international markets.

Phase three: Making decisions about entry mode choice.

Phase four: Defining global competitive advantages.

This model specifically obtains the type of information that international business decision makers consider when appraising and tailoring customized international positioning processes.

*Pharmaceutical marketing context*

As mentioned previously, the quality of pharmaceutical marketing is entirely different from that of other products. This difference is dominated by the pharmaceutical market environment which is actually considered as health care environment (17).

Patients are the main customers in pharmaceutical marketing, and health care is provided to them through supplying medicine (18).

From the customer’s point of view, pharmaceutical marketing is a multi-type customer criterion. As the final consumers of the products are individual people, it may be expected to be very similar to consumer products and core marketing principals applicable to regular consumer goods. However, it has specific unique characteristics such as the fact that decision makers for buying are not the consumers, but they are usually professionals or organizations such as physicians, hospitals, or governors who are buying influencers (18).

Health care services are not limited to supplying pharmaceutical products to patients. These services can also be prepared and delivered by various centers such as: hospitals, ambulatory care centers, long-term care facilities, sub-acute care facilities, or in-home care.

The health care network consists of patients, professionals and other related centers which, as a whole, comprise the health care system. Its major components include hospitals, health maintenance organization (HMOs), insurance companies, pharmaceutical firms, government, physicians, pharmacists, charity organizations, etc. (17).

To successfully launch pharmaceuticals in any market, it is necessary to determine and analyze the health care system of each market and design an appropriate marketing plan according to the specifications of each market.

The important differentiating factor in this industry is the tough regulatory and control systems. This is due to the need for pharmaceutical delivery to patients and its association with human safety and life (19).

There are several national and international regulatory systems and control bodies which control pharmaceutical activities on both the supply and demand side. They may focus on patent protection, registration, pricing, and manufacturing of new products (the supply-side), or may impose trade restrictions across national borders or place restrictions on marketing and prescription behavior (demand-side) (17).

*Whitelock modified model for Iran*

Whitelock model, which is basically an integration of four previously mentioned models, was found appropriate and considered as a suitable base model for Iran. The favorable position of Whitelock model over others is that it discusses, compares and contrasts all four models and finally formulates them into one single model.

As the proposed model shows, the pharmaceutical companies need to well understand the international regulatory affair environment and to identify the effective factors of pharmaceutical business in target markets in order to involve in export marketing. The influencing factors or dimensions of target markets in pharmaceutical marketing according to our findings are: Health care system, regulatory system, influencers, accessibility, competitors and potentials.

On the other hand, the internal factors that are very effective in export readiness according to the model include: orientation and philosophy, regulatory affair skills, resources, objectives and management commitment. Consequently, the pharmaceutical companies are supposed to analyze the target markets, after assessment of internal factors and ensuring their adequacy, considering essential factors and to investigate them in order to select the best market(s) to start export marketing.

Analysis of target market and matching it with company’s capabilities and conditions requires awareness of regulatory affair and export aspects. After this phase and selecting the target market(s), it can be supposed that the company has acquired export readiness and can begin export marketing by choosing the best market entry mode. 
